# Transfer of motor and perceptual skills from basketball to darts

**DOI:** 10.3389/fpsyg.2013.00593

**Published:** 2013-09-12

**Authors:** Rebecca Rienhoff, Melissa J. Hopwood, Lennart Fischer, Bernd Strauss, Joseph Baker, Jörg Schorer

**Affiliations:** ^1^Department of Sport Psychology, Institute of Sport and Exercise Sciences, University of MuensterMuenster, Germany; ^2^College of Sport and Exercise Science, Victoria University, Melbourne, VIC, Australia; ^3^School of Kinesiology and Health Science, York UniversityToronto, ON, Canada; ^4^Department of Sport and Movement Science, Institute of Sport Science, University of OldenburgOldenburg, Germany

**Keywords:** perception, expertise, quiet eye

## Abstract

The quiet eye is a perceptual skill associated with expertise and superior performance; however, little is known about the transfer of quiet eye across domains. We attempted to replicate previous skill-based differences in quiet eye and investigated whether transfer of motor and perceptual skills occurs between similar tasks. Throwing accuracy and quiet eye duration for skilled and less-skilled basketball players were examined in basketball free throw shooting and the transfer task of dart throwing. Skilled basketball players showed significantly higher throwing accuracy and longer quiet eye duration in the basketball free throw task compared to their less-skilled counterparts. Further, skilled basketball players showed positive transfer from basketball to dart throwing in accuracy but not in quiet eye duration. Our results raise interesting questions regarding the measurement of transfer between skills.

## Introduction

The importance of good free throw abilities for professional basketball players is unquestioned. In the NBA for example, the top 10 shooters of the 2011/2012 season had an average success rate of 82.4% with some achieving exceptionally high percentages (e.g., Dirk Nowitzki with 90.5%). Vickers and colleagues (Vickers, 2007, 2009) proposed that one of the reasons behind such exceptional performance is that successful shooters show a longer “quiet eye,” a phenomenon relating to a performer's gaze behavior prior to task execution. The quiet eye is defined as the final fixation (of at least 100 ms) prior to the onset of the final movement in a motor task (Vickers, 2007) and is associated with superior performance, explaining both inter- and intra-group variability (Mann et al., 2011) in a variety aiming tasks (for an overview, see Vickers, 2007). Studies of inter-group differences have shown that higher skilled athletes have a longer quiet eye duration and an earlier onset of their final fixation compared to less-skilled athletes (e.g., Vickers, 1996; Williams et al., 2002; Vickers and Williams, 2007; Causer et al., 2010), while studies of intra-group variability show longer and better timed quiet eye durations with successful compared to unsuccessful performances (e.g., Vickers, 1996; Vickers and Adolphe, 1997; Janelle et al., 2000; Harle and Vickers, 2001).

It is assumed that the quiet eye reflects a critical period of cognitive processing where relevant movement components of the ensuing response are fine-tuned and programmed, such as force, direction, and velocity (Williams et al., 2002). Moreover, the period is seen as representing the time required to organize the neural networks and visual parameters responsible for orientation and control of visual attention (Vickers, 1996). Thus, the timing of the quiet eye period is important (Vickers et al., 2000) and an optimal quiet eye period should help direct attention to the target and protect against distraction (e.g., Wilson and Pearcy, 2009). Although the quiet eye onset during the aiming movement has been shown to be important in several studies, the role of the timing is still under examination (for a review, see Vine et al., 2012). Based on the collective results of studies examining this phenomenon, having a long quiet eye duration has been emphasized for an optimal focus of visual attention (for a review, see Vine et al., 2012) and an optimal period of cognitive pre-programming (Moore et al., 2012). In addition, a longer quiet eye period may result in increased postural stability, which is important for aiming tasks (i.e., a general quiescence of the psychomotor system, Vine et al., 2011).

While researchers have provided great support for the notion and the value of the quiet eye, the extent to which this skill can transfer across similar domains is unknown. It seems reasonable to suggest transfer should occur given the proposed mechanisms of the effect (e.g., if gaze stability is important for one aiming task, it would be important for similar tasks), which has important implications for our understanding of sport skill acquisition.

Two related theories focus on “similarity” as the critical component of learning transfer (Magill, 1998). In the first theory, transfer of learning is said to occur because of similarity in skill and context, thus, the more similar skill or context components are, the higher will be the positive transfer. This is based on one of the earliest explorations of transfer of learning, where Thorndike (1914) proposed that transfer occurs between “identical elements” shared across the different tasks (*identical element theory*). Baker and Côté (2006) proposed that identical elements could include (1) physical conditioning elements, which relate to general physiological changes shared between similar modes of activity (e.g., all aerobic training will promote system-wide cardiorespiratory changes), (2) movement elements, which relate to the anatomical and biomechanical similarities between tasks (e.g., overhand throwing in baseball and handball), (3) perceptual elements, which relate to the environmental information used to make performance related decisions (e.g., the need to recognize offensive and defensive patterns of play) and (4) conceptual elements, which relate to similarities in the strategies, rules and guidelines governing behavior during competition (e.g., gymnastics and diving share some conceptual elements regarding judging aesthetics of performers' movements).

The second theory explains the existence of positive transfer as the result of similarities in learning or performance process characteristics. The *transfer-appropriate-processing* view (Lee, 1988) postulates transfer occurs due to similarities between the learning processes required for performance situations. For example, Damos and Wickens (1980) demonstrated positive transfer between training and test situations resulting from similar processing activities in both tasks, even with dissimilar task characteristics. In both theories, transfer is a complex process and several variables can influence the rate and degree of transfer (e.g., physical, temporal, social, or functional characteristics; Barnett and Ceci, 2002). Concerning the specific transfer of perceptual elements, it is important to differentiate between intra-task transfer, which refers to transfer of a skill practiced in one context to a novel context, and inter-task transfer, which relates to the influence of experience with one skill on the performance of a new skill (Magill, 1998). There has been some consideration of both intra-task (Singer et al., 1994; Starkes and Lindley, 1994; Tayler et al., 1994; Williams et al., 2003) and inter-task transfer (e.g., Smeeton et al., 2004; Abernethy et al., 2005); however, studying inter-task transfer of perceptual skill could provide deeper insight into the factors enabling expert players to apply their existing knowledge to new situations (Smeeton et al., 2004). Rosalie and Müller (2012) highlight the relevance of investigating transfer between sports tasks (i.e., not only rudimentary tasks typically used in laboratory experiments) and determining whether critical components of perceptual-motor skills transfer across domains; as they note, our understanding of how, and to what extent perceptual and perceptual-motor skill transfer occurs is still limited. Given the results of prior research identifying the transferability of visual search behavior, it is important to determine the extent of transferability of other perceptual behaviors like fixation duration across sportive tasks (Williams et al., 1999; Smeeton et al., 2004).

In particular, transferability of the quiet eye has rarely been investigated. There is some evidence of quiet eye transfer from training to competitive play within the same sportive task (i.e., intra-task transfer), as demonstrated in elite/expert performers in volleyball (Adolphe et al., 1997), basketball (Harle and Vickers, 2001), shooting (Causer et al., 2011), and golf putting (Vine et al., 2011). No studies have examined the transferability of quiet eye from one related sporting task to another (i.e., inter-task transfer). From a theoretical perspective, this might deepen our understanding of the phenomenon's specificity (e.g., performing a transfer task coming along with the same perceptual behavior as within the fundamental task refers to a more general applicability). From a practical perspective, this question explores the necessity of domain-specific perceptual training interventions, and the fruitfulness of practicing similar but not identical movements. Given the proposed mechanisms of the quiet eye effect, it seems reasonable that quiet eye (i.e., fixation duration) should transfer to similar aiming tasks.

In this investigation we examined two main hypotheses. The first concerns performance and quiet eye within a basketball free throw shooting task (fundamental task) where we hypothesized that skilled basketball players would have superior performance compared to less-skilled players. Aligned with this first hypothesis, we expected quiet eye differences for the expertise-specific task of basketball free throw shooting such that (1) there would be expertise differences with longer quiet eye duration for skilled participants in comparison to less-skilled (inter-group variability) and (2) successful performance (hits) would be associated with longer quiet eye durations than unsuccessful ones (misses) across skill levels (intra-group variability). These assumptions would replicate previous findings on expertise and the quiet eye, which is essential for the question of transfer within this context.

Our second hypothesis considered the transfer of performance and quiet eye from basketball free throw shooting (fundamental task) to dart throwing (transfer task) based on the idea of sharing identical elements (Thorndike, 1914). For example, several conceptual elements are shared between the basketball free throw and dart throw as both tasks are self-paced overhand tasks, requiring participants to propel an object in the forward direction towards a static target with self-initiated release. Both tasks have precise beginning and end points and show little variability between practice and end game performance (Keetch et al., 2008). Perhaps more importantly, previous research has highlighted similarities in movement elements between the two tasks (e.g., both have a similar duration of the motoric extension of the arm; Oudejans et al., 2002). Moreover, there may be some transfer between the horizontal and vertical elements of the movement execution. More specifically, the limiting factor for a successful free throw may relate to horizontal deviation, not vertical and, as a result, it is possible that differences between the basketball free throw task and the dart throwing task might be greater in the vertical axis (*y*-axis) than the horizontal axis (*x*-axis). This is a realistic assumption considering that in both tasks the participant is aiming in line with the center of his body on the horizontal axis, but the height of the target is different and the required trajectory of the dart/ball is different, both components related to the vertical axis.

On this basis, we hypothesized that (a) skilled basketball players would show better transfer of throwing performance, resulting in higher throwing accuracy on the dart throwing task, compared to the less-skilled basketball players despite no prior experience in darts for either group; and (b) that these differences would be reflected in smaller deviations on the horizontal axis (*x*-axis) for skilled versus less-skilled players, but similar deviations on the vertical axis (*y*-axis) for both skill groups.

Additionally, we investigated the inter-task transfer of the perceptual processes associated with quiet eye, which has not yet been examined. Given the parallel evidence for the quiet eye phenomenon in both basketball free throw shooting (Vickers, 1996; Harle and Vickers, 2001; Wilson et al., 2009; Vine and Wilson, 2011) and dart throwing (Vickers et al., 2000; Rienhoff et al., 2012), being able to fixate an aim could be an identical element as described by Baker and Côté (2006). Furthermore, the quiet eye duration is seen as a critical period of information processing within targeting tasks (e.g., Vickers, 1996) and responsive to variability of practice and axis of target changes (Horn et al., 2012). Thus, in accordance with the transfer-appropriate-processing view (Lee, 1988) the two tasks may share similar processing requirements. Second, there is some evidence for the transferability of perceptual skills like fixation duration. The specific visual search behaviors transfer from one sport task to another, expressed by similar number of fixations of equivalent duration (Williams et al., 1999; Smeeton et al., 2004). Based on these two lines of argumentation, we hypothesized transferability of the quiet eye period from the fundamental task of basketball free throw shooting to the novel transfer task of dart throwing, resulting in longer quiet eye duration for skilled basketball players compared to less-skilled players in both tasks.

## Materials and methods

### Participants

Thirteen skilled and 13 less-skilled basketball players participated in this experiment. All were male right-handers with normal or corrected-to-normal vision. Skilled players had a mean age of 24.2 years, *SD* = 4.2, and less-skilled players had a mean age of 25.0, *SD* = 2.9. The skilled players were members of a third division German basketball team with an average of 12.9 years of playing experience, *SD* = 5.0, and an average of 6.1 training hours per week, *SD* = 1.9. The less-skilled players were German university physical education students with a maximum of 3 months of experience in basketball (i.e., as part of their physical education curriculum). Participants in both groups declared no experience in dart throwing. All participants provided informed consent prior to data collection and the study was conducted in accordance with the ethical principles described in the declaration of Helsinki (World-Medical-Association, 2008).

### Apparatus and tasks

The experiment consisted of two parts, the first involved the basketball free throw, and the second involved the dart throwing task. The experiment was conducted in a laboratory setting where the tasks were accomplished following standard international basketball and dart rules.

For the basketball free throw task, a portable standard basketball hoop was utilized. The rim was 3.05 m high and the free throw line was at a distance of 4.23 m to the middle of the hoop, which are both standard measures according to international basketball rules by the International Basketball Federation (FIBA). For the dart throwing task, the height of the target center was 1.73 m and participants stood 2.37 m away from the board; again, these are the standard measures according to international dart rules from the World Darts Federation (WDF). In order to get accurate measurements of the horizontal and vertical axis, instead of a regular dart board, a wooden board with a specific squared raster was presented. A squared field in the middle of the raster, representing the bull, had a size of 1.5 × 1.5 cm including the bull's eye of 0.5 × 0.5 cm. Fifteen horizontal and vertical lines separated by a distance of 0.5 cm were drawn around the bull creating a 31 × 31 cm matrix of 0.5 cm squares.

For eye movement analyses, a light-weight head-mounted eye tracking system was used (Arrington Research BS007, Inc. Scottdale, AZ, USA). Scene-related eye-positions were stored using an additional 0.2 MB head-mounted scene camera at a frequency of 30 Hz, an accuracy of 0.25–1.0°, and a resolution of 0.15°. The eye tracking system runs with View Point PC 60 (Version 2.8.3.0) and was linked to the host computer (AMD Athlon 64, Processor 3500+; 1 GB RAM) via a 10-m cable. This setup allowed all participants to move freely.

Within both tasks, an externally positioned digital video camera (CASIO EX-F1 EXILIM) was placed 2.5 m to the left of the participant, presenting the participants' sagittal plane. The view showed the participants' pre-performance routine and throwing action for the basketball free throw and dart throwing performances. Prior to each task, a light was presented to the two different camera systems. This procedure ensured that a clear event could be used for synchronization of the two camera systems for subsequent offline analysis. This procedure is similar to the perception-action-coupling using split-screen images, as used in other quiet eye studies (Vickers, 2007).

### Measures of performance

For the basketball free throw task, shooting accuracy was scored as hit without rim contact (4 points), hit with board or rim contact (3 points), miss (2 points) or air ball (1 point). We utilized this four level measure in accordance with earlier free throw studies (Wulf et al., 2005; Zachry et al., 2005). The more detailed measurement thereby increased the reliability of the skill differences (Vaughn et al., 2012). Participants used an official regulation-sized FIBA basketball (Spalding, size 7). For the dart throwing task, throwing accuracy was measured using mean radial error, which captures the mean of error's magnitude in 2-D using the *x* and *y* coordinates of each shot (radial error). This involved analyzing the radical of the squared deviations in the horizontal (*x*-axis) and vertical (*y*-axis) directions from the bull's eye (Hancock et al., 1995). The deviation measurement was represented by squares from −15 to +15, relative to the bull's eye (0/0). Thus, after every single throw, first the value for the *x*-axis was captured, followed by the value for the *y*-axis. These two values were the basis for the mean radial error. Participants used competition steel darts with a weight of 24 g. It is noteworthy to point out that basketball free throw shooting was scored using an upward directed scale (better performance was represented by higher score), while dart throwing used mean radial error reflecting a downward directed scale where better performance was characterized by lower score.

### Measures of quiet eye duration

As defined earlier, the quiet eye is the last fixation prior to the initiation of the final movement towards the target. Both throwing tasks were divided into three different phases. The dart throwing movement contained alignment, flexion and extension phases of the throw (Vickers et al., 2000); the basketball free throw contained a preparation, a ball-up and an extension phase (Vickers, 2007). For both movements, the period of quiet eye was seen as the final fixation towards the target prior to the initiation of the extension phase. More precisely, the location of the final fixation was on the rim or backboard in basketball free throw shooting or on the raster in dart throwing for at least 100 ms with a saccadic threshold of 0.1. This can be operationalized by the last video frame of the external camera footage before the angle between the upper and lower arm starts to increase, as the throwing arm moves towards the target (Vickers et al., 2000; Vickers, 2007; Wilson et al., 2009).

### Procedure

Prior to beginning, participants were given basic information about the experiment and completed a questionnaire which gathered their age and level of experience in basketball. Before data collection, an individual adjustment of the eye-tracking system was implemented and calibrated. For both tasks, monocular vision of the eye on the throwing arm side was captured. The wooden dart board was set up underneath the basketball ring to allow for a brief switch between the two tasks. For the dart throwing task, participants stepped forward from the free throw line to a marker indicating the correct distance for the dart throw. The order of basketball free throwing and dart throwing tasks was presented counterbalanced. Within each task, participants took 15 throws. After every trial, performance accuracy was recorded. For the basketball free throw, performance was assessed according to the different levels described above. For dart throwing, the deviation of the dart on the *x*- and *y*-axes after every trial was gathered.

### Statistical analysis and dependent variables

Statistical analyses were conducted separately for the basketball (fundamental task) and dart (transfer task) tasks. Furthermore, throwing performance and gaze behavior were considered separately. For throwing accuracy in the basketball free throw task, a one tailed *t*-test for independent samples was used to analyze skill differences between skilled and less-skilled players. For quiet eye duration in the basketball task, a one-tailed *t*-test for independent samples was conducted for the intergroup variability; and a *t*-test for dependent samples for intragroup variability.

For throwing accuracy and quiet eye duration in the transfer task of dart throwing, a 2 (skill: skilled vs. less-skilled) × 2 (task: basketball vs. darts) ANOVA was performed. As postulated previously, performance scales for basketball and dart were incomparable using the raw data. Consequently, *a posteriori z*-transformations were conducted for comparison and ANOVAs were analyzed on this basis of *z*-scores. *Z*-transformations are used to make differences between two varying dependent variables comparable on the basis of means and standard deviations; thus, *z*-scores are standardized values helping to express different values (c.f. for basketball and dart performance) in a common scale; calculated by using the original value minus the mean of the distribution divided by its standard deviation. By calculating *z*-scores, data from basketball free throw shooting and dart throwing can be compared (for a detailed explanation, see Moore and McCabe, 2003). Moreover, effect sizes (*d* for *t*-test and *f* for ANOVA) and test power (1 − β) were calculated in accordance with Cohen (1988). The alpha-level was set to 0.05 and all data analyses were conducted using SPSS 20.0 and G^*^Power 3.1.10 (Faul et al., 2007).

## Results

In hypothesis one, we assumed greater throwing accuracy for skilled compared to less-skilled basketball players in the expertise-specific task of basketball free throw shooting. A one-tailed *t*-test for independent samples revealed a significant difference between skilled and less-skilled players, *t*_(24)_ = 9.21, *p* < 0.01, *d* = 3.61. As can be seen in Table 1, skilled players outperformed the less-skilled.

**Table 1:** **Comparison of throwing performance (basketball, scale 1–4; darts, mean radial error) and quiet eye duration (QE, in seconds) in basketball (additionally separated by hits and misses) and darts differentiated by skill groups (*M* = mean; *SE* = standard error)**.

	**Less-skilled**	**Skilled**
Basketball	*M* = 2.39, *SE* = 0.05	*M* = 3.23, *SE* = 0.07
Darts (MRE)	*M* = 11.23, *SE* = 0.50	*M* = 8.78, *SE* = 0.66
QE Basketball	*M* = 1.19, *SE* = 0.09	*M* = 1.44, *SE* = 0.13
QE Basketball hits	*M* = 1.17, *SE* = 0.10	*M* = 1.44, *SE* = 0.15
QE Basketball misses	*M* =1.18, *SE* = 0.09	*M* = 1.47, *SE* = 0.12
QE Darts	*M* = 3.80, *SE* = 0.43	*M* = 3.79, *SE* = 0.40

For the perceptual performance during the basketball free throw task, quiet eye duration was analyzed; here we hypothesized (a) longer quiet eye durations for more skilled players (intergroup variability) and (b) that hits come along with longer quiet eye durations than misses (intragroup variability). For the intergroup variability, a one-tailed *t*-test for independent samples was conducted, *t*_(24)_ = −1.53, *p* = 0.07, *d* = 0.60, 1 − β = 0.44. From the perspective of effect-size (Cohen, 1988) this can be considered a medium-sized effect, which is in accordance with previous quiet eye studies (c.f. Rienhoff et al., 2012). For the intragroup variability, *t*-test for dependent samples revealed non-significant differences for hits and misses with low effect sizes, *t*_(24)_ = −0.43, *p* = 0.67, *d* = 0.05, 1 − β = 0.06.

Second, the results of hypothesis two concerning the transfer of expertise from basketball to darts are presented. In order to compare data between the tasks, we analyzed transfer of skills from basketball to darts on the basis of *z*-scores, both for throwing results and for quiet eye duration. For throwing accuracy, ANOVA revealed significant differences between groups, *F*_(1, 24)_ = 46.71, *p* < 0.01, *f* = 1.40 and a significant interaction of both factors, *F*_(1, 24)_ = 3.57, *p* = 0.04, *f* = 0.39, but no significant differences for task, *F*_(1, 24)_ = 0.00, *p* = 1.0 (Due to technical reasons (i.e., standardized *z*-scores which have the same mean of zero for both tasks) these tests cannot reveal differences. Consequently, within subject effects for the two ANOVAs relating to hypothesis two were only reported for the sake of completeness). Consequently, skilled players outperformed the less-skilled not only on the domain-specific task (i.e., basketball), but also in the novel transfer task (i.e., darts).

Moreover, we hypothesized that skill group differences would be more strongly reflected in differences on the horizontal axis, while vertical axis deviation would be similar for both skill groups. Consequently, to explore whether a specific part of the radial error explained the significant differences, we analyzed the errors on the *x*- and the *y*-axis separately. Although the skilled basketball players outperformed the less-skilled players on both axes, a two-tailed *t*-test for independent samples revealed a significant difference between the groups for the *x*-axis only, *x*-axis: *t*_(24)_ = 2.23, *p* = 0.03, *d* = 0.87; *y*-axis: *t*_(24)_ = 1.42, *p* = 0.17, *d* = 0.56, 1 − β = 0.39. For quiet eye duration, a different pattern was revealed; neither skill differences, *F*_(1, 24)_ = 1.21, *p* = 0.28, *f* = 0.23, 1 − β = 0.25, nor task differences, *F*_(1, 24)_ = 0.00, *p* = 1.0, nor the interaction of both factors were significant, *F*_(1, 24)_ = 1.03, *p* = 0.32, *f* = 0.20, 1 − β = 0.52. Consequently, in the basketball free throw task, skilled players had longer quiet eye duration, but these differences were not statistically significant in the transfer task.

## Discussion

In the current investigation we aimed to replicate the findings that highly skilled performers have longer quiet eye durations in basketball shooting than lower skilled performers and that this longer fixation duration is associated with better free throw performance (Vickers, 1996). Furthermore, and more importantly, we investigated whether this superiority can be transferred to another task (i.e., dart throwing). We hypothesized that skilled basketball players would outperform less-skilled players in the expertise-specific task of basketball free throw shooting, showing a higher throwing accuracy and longer quiet eye duration. Besides that, we assumed longer quiet eye duration for hits in comparison to misses.

Our throwing results supported the expected differences between the skill groups; skilled basketball players significantly outperformed the less-skilled in the basketball free throw task. Moreover, analysis of perceptual performance for this task, represented by the duration of quiet eye, identified differences in gaze behavior between skilled and less-skilled players. The skilled basketball players showed longer quiet eye duration than the less-skilled. Surprisingly, we were unable to replicate significant quiet eye duration differences on the basis of *p*-value (similar to Janelle et al., 2000; Rodrigues et al., 2002). This could have resulted from variability in the shooting styles between the skill groups; for instance, all skilled but only two less-skilled participants used a high shooting style (i.e., ball and hand carried above the head until ball release after extension of the elbow; participant can look at the basket from underneath the ball), while the rest of the less-skilled group used a low shooting style (i.e., ball and hands remain below eye level prior to extension of the elbow, thereafter they move in front of the face). Research suggests that the type of information processes that take place and the information used during the quiet eye phase differs between shooting styles (de Oliveira et al., 2008), which might confound comparisons of quiet eye durations between the skill groups (de Oliveira et al., 2006). However, if we consider the effect sizes and test power (as recommended by APA, 2010), the effect size of the between group quiet-eye differences (0.60) represented a medium sized effect in accordance to Cohen (1988). The size of this effect emphasizes the impact of quiet eye differences and is in accordance to previous results (for a meta-analysis, see Mann et al., 2011). We do not want to argue that effect sizes should negate the importance of significant *p*-values, but would argue that the current results are in line with previous quiet eye findings (for deeper explanation, see Rosnow and Rosenthal, 1989). Consequently, the differences between skill groups in quiet eye duration observed in previous investigations were successfully replicated in our experiment. However, previous studies have also identified an association between quiet eye duration and performance, with hits involving longer quiet eye durations compared to misses (Vickers, 1996; Causer et al., 2010); these results were not replicated in our study. It is possible that differences between the current investigation and prior work were due to a methodological difference to previous investigations. We did not use a fixed trial count of hits and misses, like previous studies (e.g., 10 hits vs. 10 misses, Vickers, 1996), but analyzed the real distribution of hits and misses within the 15 free throws. As the skilled players performed a higher proportion of hits, and the less-skilled players a higher proportion of misses, the results were influenced by the small number of misses for skilled and hits for less-skilled. Thus, the *t*-test could have been affected by the differences between hits vs. misses in the skill groups compared in the current study, which was not an issue in previous research. Together, these results replicate previous findings of superior free throw performance and longer quiet eye duration for highly skilled performers in comparison to less-skilled counterparts; however, hits were not associated with longer quiet eye durations than misses.

Our second objective was to investigate the extent to which expertise in basketball free throw shooting can be transferred to dart throwing. We hypothesized differences between the skill groups in the dart throwing task for both throwing accuracy and quiet eye duration. With regard to throwing accuracy, the skilled basketball players outperformed the less-skilled players significantly, although both groups had no previous training in this task. This difference suggests that transfer in throwing performance occurred potentially due to the similar complex movement patterns in the two different tasks (Weiss et al., 2008). More precisely, we hypothesized that transfer occurs based on horizontal axis differences, which was reflected in the dart throwing results. The skilled players showed significantly smaller deviations on the *x*-axis (horizontal axis) compared to the less-skilled players, while the *y*-axis deviation (vertical axis) was similar for both skill groups. This suggests that the skilled participants used the movement patterns they were familiar with from basketball, whereas the less-skilled were not equipped to do so. It appears that the skill of keeping a throwing object like a ball in line with the center of your body in basketball is more likely to transfer to keeping the dart in line with the center of your body, but adjusting to the vertical changes might be more novel and, as a result, more difficult. Thus, it seems that positive transfer occurred based on the similar components, as suggested by identical elements theory (Thorndike, 1914; Schmidt and Wrisberg, 2000; Baker and Côté, 2006).

We also hypothesized transferability of the quiet eye period from the basketball free throw task to dart throwing, resulting in longer quiet eye durations for skilled versus less-skilled basketball players in the transfer task. Here, our results did not support our hypothesis; there were almost no differences in the duration of the quiet eye period between skilled and less-skilled basketball players on the transfer task. Thus, in line with the hypothesis of identical elements (Thorndike, 1914) and even in accordance with the transfer-appropriate-processing view (Lee, 1988) of sharing similar processing requirements via quiet eye duration, no transfer of perceptual components occurred between the two tasks. This is an unexpected and interesting finding, because it questions again the underlying mechanism of the quiet eye (e.g., see Rienhoff et al., 2012). It is possible that the phenomenon of quiet eye is highly domain specific and transfer to similar aiming tasks does not occur. The longer quiet eye durations used by skilled participants in their expertise-specific task did not seem to be utilized within the novel transfer task, which challenges its necessity for performing the task. Moreover, it can be postulated that the quiet eye is a product of good performance, being displayed only once a skill has been learned to a certain degree (e.g., quiet eye in training interventions, for a review, see Vine et al., 2012). Concerning its similarity, the perceptual components of basketball free throw shooting and dart throwing seemed to be dissimilar enough that processing requirements could not be transferred. This notion should be investigated in further research in order to give further insight into the transferability of the quiet eye.

Based on these findings it is difficult to derive explicit practical implications. Concerning the motor performance, transfer occurred between these tasks suggesting that participation in similar skills may augment development of performance in aiming sports [similar to the “sampling” position advocated by Côté (1999) and others]. However, the perceptual results, suggest that explicit inter-task transfer of quiet eye does not occur suggesting that “deliberate practice” (i.e., highly specialized, task-specific training; Ericsson et al., 1993) may be most relevant for the development of this type of skill. However, it should be clear that recommending detailed and valid training implications on the basis of these results is highly speculative without further investigation exploring the interplay of motor and perceptual components. Within this context, it should be acknowledged that the underlying mechanism of transfer is likely unique to the individual and the transfer event/domain (Rosalie and Müller, 2012) and that transfer comprises a continuum of several variables influencing its outcome (Barnett and Ceci, 2002). Additionally, further factors like motivation and fatigue might influence transfer (Rosalie and Müller, 2012).

In sum, this study replicated differences between skill groups in throwing accuracy and quiet eye duration in the expertise-specific task of basketball free throw shooting. Moreover, our results suggest that transfer occurred on a specific component of throwing performance, namely the skill to throw straight on the *x*-axis, a factor that may be task dependent. Within this study, we used a squared raster as the target for the dart task to allow for optimal capturing of mean radial error. It is possible that the vertical deviation becomes more sensitive when throwing to a normal (i.e., round) dartboard. For example, a miss on the *y*-axis might cause the miss of triple 20, while slight left and right deviations on the *x*-axis might still hit the field.

It is also possible that the similarity between basketball free throw shooting and dart throwing is not as high as assumed. Even if the tasks share several elements, there are still many qualitative and quantitative task specific and technical characteristics. As mentioned before, the two tasks might be so dissimilar that the specific perceptual components examined in this study do not transfer. Further, there could be an interaction between several factors impairing the quiet eye period (e.g., practice time or motor synergies). Further investigation should also explore whether the transferability is one-sided from big to fine motor skills (from basketball to darts) or whether skilled dart players also outperform less-skilled dart players in basketball free throwing. This could shed light on the issue of how similar these tasks really are. Additionally, further research might also consider a more specific motor transfer based on kinematic analyses of movement patterns across similar domains. This would lead to deeper knowledge about similarities between different movements. For the perceptual skill of quiet eye, no transfer was revealed; the significant differences in the expertise specific task of basketball free throw shooting diminished in the transfer task leading to further questions concerning the transferability of perceptual skills between sport tasks.

### Conflict of interest statement

The authors declare that the research was conducted in the absence of any commercial or financial relationships that could be construed as a potential conflict of interest.
